# Draft genome sequences of four *Achromobacter ruhlandii* strains isolated from cystic fibrosis patients

**DOI:** 10.1590/0074-02760160130

**Published:** 2016-10-31

**Authors:** Elenice RA Rodrigues, Géssica A Rocha, Alex G Ferreira, Robson S Leão, Rodolpho M Albano, Elizabeth A Marques

**Affiliations:** 1 Universidade do Estado do Rio de Janeiro, Faculdade de Ciências Médicas, Departamento de Microbiologia, Imunologia e Parasitologia, Rio de Janeiro, RJ, Brasil; 2 Universidade do Estado do Rio de Janeiro, Instituto de Biologia Roberto Alcântara Gomes, Departamento de Bioquímica, Rio de Janeiro, RJ, Brasil

**Keywords:** Achromobacter ruhlandii, genome sequence, cystic fibrosis

## Abstract

*Achromobacter* species are being increasingly isolated from the respiratory tract of cystic fibrosis patients. Recent reports indicate that *Achromobacter ruhlandii* is a potential human pathogen in cystic fibrosis-related infections. Here we report the draft genome of four *A. ruhlandii* strains isolated from cystic fibrosis patients in Brazil. This report describes *A. ruhlandii* as a potential opportunistic pathogen in cystic fibrosis and provides a framework to for additional enquires into potential virulence factors and resistance mechanisms within this species.

*Achromobacter ruhlandii* is a Gram-negative bacterium naturally found in soil (Packer & Vishniac 1955). However, recent reports indicate that *A. ruhlandii* is a potential human pathogen in cystic fibrosis-related infections (Ridderberg et al. 2012, Spilker et al. 2012a). A PAN-resistant *Achromobacter* clone, designated the danish epidemic strain (DES), causing infection in cystic fibrosis patients in Copenhagen (Hansen et al. 2006) and Aarhus (Ridderberg et al. 2011), was recently identified by multilocus sequence typing (MLST) as *A. ruhlandii* (Ridderberg et al. 2012) *. A. ruhlandii* has also been reported as the second most commonly isolated *Achromobacter* species from cystic fibrosis patients (Spilker et al. 2012b).

Here we describe draft genome sequences of four *A. ruhlandii* strains isolated from sputum of Brazilian cystic fibrosis patients attended at Instituto Nacional da Saúde da Mulher, da Criança e do Adolescente Fernandes Figueira (IFF-FIOCRUZ) and Hospital Universitário Pedro Ernesto (HUPE-UERJ), in 2007 and 2008. The isolates were identified to species level by sequencing seven housekeeping genes that were subsequently submitted to the *Achromobacter* MLST database where they were assigned to specific STs (Spilker at al. 2012a; http://pubmlst.org/achromobacter/). Furthermore, the species specific marker genes for *A. xylosoxidans* (*bla*_OXA-114_) and for *A. ruhlandii* (*bla*_OXA-258_) were amplified and sequenced to confirm species assignment (Turton et al. 2011, Papalia et al. 2013). Accordingly, the sequences from all four strains showed identity with *bla*_oxa-258_.Strains 6241, 7863, 7022 and 8173 were assigned STs 35, 204, 36 and 35, respectively. ST 35 was the only one shared between the two study centers. Minimal inhibitory concentration against ceftazidime, ciprofloxacin, imipenem and trimetoprim/sulphametoxazol was determined with the E-test strip (AB Biodisk, Solna, Sweden). The four samples were susceptible to antibiotics with the exception of strain 7022 that was resistant to trimethoprim/sulfamethoxazole.

Genomic libraries were constructed by transposon tagmentation with the Nextera XT DNA Library Prep kit (Illumina Inc, USA). Sequencing was performed for each isolate with the 500 cycle MiSeq Reagent v2 kit on a MiSeq benchtop instrument (Illumina). Paired-end sequence reads obtained for each of the isolates ranged from 2,017,226 to 3,232,222. Reads were corrected and assembled *de novo* into scaffolds with Spades 3.5 genome assembler (Bankevich et al. 2012). The Rapid Annotation using System Technology (RAST) v.2.0 server (http://rast.nmpdr.org) was used for general genome annotation and the following databases were used to refine RAST results: PHAge search tool (PHAST) (http://phast.wishartlab.com/), IS Blast Server (IS FINDER) (https://www-is.biotoul.fr/) and Antibiotic Resistance Genes Database-ARDB (http://ardb.cbcb.umd.edu/). The resulting scaffolds per isolate ranged from 89-111 with an average genome size of 6,481,38 bp (ranging from 6,289,667 to 6,686,778) and 56 or 58 RNA genes. The results of these analyses are summarised on Table I along with their GenBank accession numbers.

**TABLE I:** Overview of genome sequence assemblies

Strain	Hospital	Total of reads (nº)	Contigs (nº)	Genome size (bp)	RNA genes (nº)	Accession (nº)
6241 (ST 35)	IFF-FIOCRUZ	2,017,226	91	6,686,778	58	LVKM00000000
7863 (ST 204)	HUPE-UERJ	2,849,474	111	6,450,125	56	LVKO00000000
7022 (ST 36)	IFF-FIOCRUZ	3,232,222	89	6,498,950	56	LVKN00000000
8173 (ST 35)	HUPE-UERJ	2,550,870	90	6,289,667	58	LVKP00000000

HUPE-UERJ: Hospital Universitário Pedro Ernesto - Universidade do Estado do Rio de Janeiro; IFF-FIOCRUZ: Instituto Nacional da Saúde da Mulher, da Criança e do Adolescente Fernandes Figueira - Fundação Oswaldo Cruz.

The four *A. ruhlandii* strains were compared with the genome of *A. xylosoxidans* NH-44784-1996 (Jakobsen et al. 2013), an isolate from a cystic fibrosis patient. The genes involved in pathogenicity were identified, according to the annotation obtained in the RAST server and are summarised on Table II.

**TABLE II:** Identified genes in *Achromobacter ruhlandii* involved in pathogenicity

Product	Gene name	*A. xylosoxidans* NH44784-1996	AR 6241	AR 7022	AR 7863	AR 8173
Type II						
General secretion pathway	Type C,D,E,F,G,H,I,J,K,L,M,N	+	+	+	+	+
Type III						
Outer membrane pore forming protein	YscC,MxiD,HrcC, InvG	+	+	+	+	+
Inner membrane protein	YscU,SpaS,EscU,HrcU,SsaU	+	+	+	+	+
Inner membrane protein	YscT,HrcT,SpaR,EscT,EpaR1	+	+	+	+	+
Inner membrane protein	YscS	+	+	+	+	+
Inner membrane protein	YscR,SpaR,HrcR,EscR	+	+	+	+	+
Inner membrane protein	YscQ	+	+	+	+	+
Spans bacterial envelope protein	YscO	+	-	-	-	-
Cytoplasmic protein	YscL	+	+	+	+	+
Putative type III secretion protein	-	+	-	-	-	-
Bridge between inner and outer membrane lipoprotein	YscJ,HrcJ,EscJ, PscJ	+	+	+	+	+
Chaperone protein for YopD	SycD	+	+	+	+	+
Cytoplasmic LcrG inhibitor	LcrV	+	-	-	-	-
Inner membrane channel protein	LcrD,HrcV,EscV,SsaV	+	+	+	+	+
Type VI						
ClpB protein	ClpB	+	+	+	+	+
IcmF-related protein	IcmF	+	+	+	+	+
Protein ImpG/VasA	ImpG	+	+	+	+	+
Sigma-54 dependent transcriptional regulator	-	+	+	+	+	+
Uncharacterized protein ImpA	ImpA	+	+	+	+	+
Uncharacterized protein ImpB	ImpB	+	+	+	+	+
Uncharacterized protein ImpC	ImpC	+	+	+	+	+
Uncharacterized protein ImpD	ImpD	+	+	+	+	+
Uncharacterized protein ImpF	ImpF	+	+	+	+	+
Uncharacterized protein ImpH/VasB	ImpH	+	+	+	+	+
Uncharacterized protein ImpJ/VasE	ImpJ	+	+	+	+	+
VgrG protein	VgrG	+	-	-	-	-
Type VII						
Sigma-fimbriae chaperone protein	-	+	+	+	+	+
Sigma-fimbriae tip adhesin	-	+	+	+	+	+
Sigma-fimbriae usher protein	-	+	+	+	+	+
Adhesion						
PGA outer membrane secretin	PgaA	+	+	+	+	+
PGA synthesis deacetylase	PgaB	+	+	+	+	+
PGA synthesis N-glycosyltransferase	PgaC	+	+	+	+	+
PGA synthesis auxiliary protein	PgaD	+	+	+	+	+

-: refers to the ausence of gene; +: refers to the presence of gene; AR: *A. ruhlandii.*

Genes responsible for resistance to antibiotics (marC, macA macB, mexI, mexD, mexA, mexB, OprM, mexX, cmeA, cmeB, cmeC, *bla*_OXA258_) were annotated, however, only strain 7022 showed the presence of SHV-5a and APH(3’)-II. Furthermore, we also observed two resistance genes that are usually associated with mobile elements, sul1 and dfra26. However, in these genomes they could be not associated with these elements, being randomly located in the chromosome (Antunes et al. 2004, Miranda et al. 2004, Garza-Ramos & Romero 2007, Grape et al. 2007). A comparison of our *A. ruhlandii* samples with other genomic sequences of different species found in the databases demonstrated the presence of IS and transposable elements that were related to ISBcen18 (*Burkholderia cenocepacia* J2315), ISPa43 (*Pseudomonas aeruginosa*), TnAs2 (*Aeromonas salmonicida*), TnAs3 (*Aeromonas salmonicida* subsp. salmonicida A449 plasmid 4), ISRme12 (*Ralstonia metallidurans* CH34), ISBmu5 (*Burkholderia multivorans* ATCC 17616), ISBcen10 (*Burkholderia cenocepacia* J2315), ISStma15 (*Stenotrophomonas maltophilia* K279a), ISPst3 (*Pseudomonas stutzeri* OM1), IS408 (*Burkholderia cenocepacia* ATCC17616),ISPa38 (*Pseudomonas aeruginosa* DK2), ISPa39 (*Pseudomonas aeruginosa* DK2), ISPa40 (*Pseudomonas aeruginosa* DK2), ISBcen23 (*Burkholderia cenocepacia* HI2424), IS1474 (*Pseudomonas alcaligenes* ATCC14094 / *Pseudomonas alcaligenes* NCIB9867 P25X / *Pseudomonas putida* NCIB9869 P35X) and IS1162 (*Pseudomonas fluorescens* ST plasmid pEG). This illustrates the potential ability of *A. ruhlandii* to carry genetic and transferable elements that could contribute to the dissemination/acquisition of antimicrobial resistance mechanisms.

Five intact phages (PHAGE-Burkho-phi644-2-NC-009235, PHAGE-Burkho-KS14-NC-015273, PHAGE-Erwini-phiEt88-NC-015295, PHAGE-Pseudo-YMC11/02/R656-NC-028657 and PHAGE-Burkho-Bcep176-NC-007497) and five incomplete prophage regions (PHAGE-Salmon-SEN34-NC-028699, PHAGE-Burkho-BcepB1A-NC-005886, PHAGE-Burkho-BcepC6B-NC-005887,PHAGE-Entero-fiAA91-ss-NC-022750, PHAGE-Yellow-1-NC-028112) were also detected in our *A. ruhlandii* strains (Table III).

**TABLE III:** Intact phages and incomplete prophages regions identified in *Achromobacter ruhlandii* strains

Phages / incomplete prophage regions	Strain			
Intact phages	6241	7022	7863	8173
PHAGE-Burkho-phi644-2-NC-009235	+	+	+	+
PHAGE-Burkho-KS14-NC-015273	-	+	-	-
PHAGE-Erwini-phiEt88-NC-015295	-	-	+	-
PHAGE-Pseudo-YMC11/02/R656-NC-028657	-	-	-	+
PHAGE-Burkho-Bcep176-NC-007497	-	-	-	+
Incomplete prophage regions				
PHAGE-Salmon-SEN34-NC-028699	+	+	+	+
PHAGE-Burkho-BcepB1A-NC-005886	-	+	-	-
PHAGE-Burkho-BcepC6B-NC-005887	-	+	-	-
PHAGE-Entero-fiAA91-ss-NC-022750	-	+	-	-
PHAGE-Yellow-1-NC-028112	-	-	+	-

+: refers to the presence of these intact phages or incomplete prophages regions in strains; -: refers to the absence of intact phages or incomplete prophage regions in strains.

This whole Genome Shotgun project has been deposited at DDBJ/ENA/GenBankunder the accession LVKM00000000, LVKO00000000, LVKN00000000 and LVKP00000000. The version described in this paper is version LVKM01000000, LVKO01000000, LVKN01000000 and LVKP01000000.
